# P-2331. A Pilot Study to Establish a Nursing Home Syndromic Surveillance Network

**DOI:** 10.1093/ofid/ofae631.2483

**Published:** 2025-01-29

**Authors:** H Edward Davidson, Lisa Han, Kevin McConeghy, Yasin Abul, Ivis Perez, Evan Dickerson, Laurel Holland, Tiffany Wallace, Kazi T Wali, Clare M Nugent, Mandi E Winkis, Olajide Olagunju, Oladayo A Oyebanji, Debbie Keresztesy, Nicholas Sundheimer, David Canaday, Stefan Gravenstein

**Affiliations:** Insight Therapeutics, LLC, Norfolk, Virginia; Insight Therapeutics, LLC, Norfolk, Virginia; COIN-LTSS, Providence Veterans Affairs Medical Center, Providence, Rhode Island; Brown University, Providence, Rhode Island; Insight Theraputics, Norfolk, Virginia; Rhode Island Hospital, Providence, Rhode Island; Rhode Island Hospital, Providence, Rhode Island; Rhode Island Hospital, Providence, Rhode Island; Rhode Island Hospital, Providence, Rhode Island; Rhode Island Hospital, Providence, Rhode Island; Rhode Island Hospital, Providence, Rhode Island; Case Western Reserve University, Cleveland, Ohio; CWRU, Cleveland, Ohio; CWRU, Cleveland, Ohio; Case Western Reserve University, Cleveland, Ohio; VA Northeast Ohio Healthcare System, Cleveland, Ohio; Brown University, Providence, Rhode Island

## Abstract

**Background:**

Local viral surveillance data available from the CDC for nursing home (NH) staff to assess risk for acute respiratory infections (ARI) lags the occurrence of infections by ≥2 weeks. Also, the CDC report represents a minority of NHs nationally. We evaluated a pragmatic approach to expand and improve respiratory virus testing in NHs.

**Figure:**
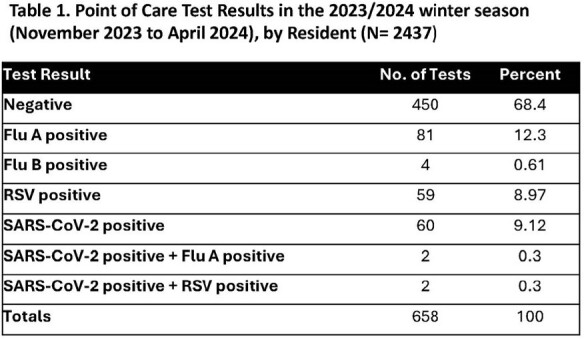

**Methods:**

We evaluated the implementation of a CLIA-waived point of care (POC) multiplex assay to assess symptomatic viral ARI as part of routine care in a NH network of typically-sized facilities (∼100 beds). The device automatically generated real time reports for participating NHs. We tested a framework for data collection and reporting to describe the natural history of ARI and expand our understanding of the burden of these infections in NHs through facility surveys.

The CLIA-waived Cepheid GeneXpert Xpress device was chosen for 4 pathogen testing due to its 1) timely result reporting (e.g., less than 30 minutes) for immediately actionable results, 2) ability to use a nasal swab, a process familiar to key NH staff, 3) ability to allocate data fields on the touch screen for the entry of patient symptoms at the time of testing, for syndromic surveillance, and 4) automatic upload of de-identified data to the cloud providing the ability to report anonymized results to a network dashboard while retaining facility site location. Data collected between November 2023 and April 2024 are presented.

**Results:**

We enrolled 22 NHs in 4 of 10 HHS reporting regions, and placed a Cepheid GeneXpert Xpress device in each, with results reporting in real time to a central database. A high proportion of tested specimens (38%) confirmed viral infection (Table 1). We found the barriers to setting up the network included unfamiliarity with the technology, identifying space to place the device, staff training, getting the device network access, addressing cybersecurity concerns, ensuring symptom entry by NH staff, and NH staff turnover. According to facility surveys, the NH staff universally liked that access to the device produced immediate, actionable, and highly sensitive and specific results for RSV, SARS-CoV-2, and influenza A and B.

**Conclusion:**

CLIA-waived viral ARI POC testing can be implemented in NHs and identified SARS-CoV-2, influenza, or RSV in about 30% of all samples.

**Disclosures:**

H. Edward Davidson, PharmD, MPH, GSK: Grant/Research Support|Moderna: Grant/Research Support Lisa Han, MPH, GSK: Grant/Research Support|Moderna: Grant/Research Support Kevin McConeghy, Pharm.D., Genentech: Grant/Research Support|GlaxoSmithKline: Grant/Research Support|Moderna: Grant/Research Support|Sanofi-Pasteur: Grant/Research Support|Seqirus: Grant/Research Support Yasin Abul, MD, Moderna: Grant/Research Support|Moderna, Abt, CDC: Grant/Research Support Ivis Perez, MPH, LPN, Moderna: Grant/Research Support David Canaday, MD, Moderna: Grant/Research Support|Pfizer: Grant/Research Support Stefan Gravenstein, MD, MPH, CDC: Advisor/Consultant|CDC: Grant/Research Support|Genentech: Advisor/Consultant|Genentech: Grant/Research Support|Genentech: Honoraria|GlaxoSmithKline: Advisor/Consultant|GlaxoSmithKline: Grant/Research Support|GlaxoSmithKline: Honoraria|Janssen: Advisor/Consultant|Janssen: Grant/Research Support|Janssen: Honoraria|Moderna: Advisor/Consultant|Moderna: Grant/Research Support|Moderna: Honoraria|NIH: Grant/Research Support|Pfizer: Advisor/Consultant|Pfizer: Grant/Research Support|Pfizer: Honoraria|Sanofi: Advisor/Consultant|Sanofi: Grant/Research Support|Sanofi: Honoraria|Seqirus: Advisor/Consultant

